# High-sensitivity C-reactive protein as a better predictor of post-thrombolytic functional outcome in patients with previous antiplatelet therapy

**DOI:** 10.1186/s40001-022-00705-z

**Published:** 2022-06-03

**Authors:** Tan Li, Qiannan Yu, Yiqing Wang, Xiuying Cai, Yan Kong, Hongru Zhao, Shanshan Diao, Yiren Qin, Qi Fang

**Affiliations:** Stroke Center, The First Affiliated Hospital of Soochow University, Suzhou, 215006 China

**Keywords:** hsCRP, mRS, rt-PA, NIHSS, Antiplatelet

## Abstract

**Background:**

C-reactive protein (CRP) is an important biomarker of inflammation and plays a pivotal role in predicting the clinical prognosis of cardiovascular and cerebrovascular diseases. However, the mechanism of inflammation influencing the outcome of patients with ischemic stroke are unknown.

**Aims:**

We aim to investigate the association between hsCRP and mRS in 194 eligible patients by therapy-stratified analyses.

**Methods:**

The modification effects of antiplatelet therapy on the association between mRS and different exposure variables were analyzed. The retained variables were analyzed in the receiver operating characteristic (ROC) curve to discriminate patients with poor outcome.

**Results:**

hsCRP was positively correlated with mRS in therapy-stratified analyses. There was a statistical modification effect of antiplatelet therapy on the association of hsCRP and mRS (*P* for interaction = 0.0101). The discriminative effect of poor outcome was further verified by ROC curve analyses (AUC_with_ from 0.758 to 0.872, AUC_without_ from 0.709 to 0.713).

**Conclusions:**

hsCRP is correlated with the clinical outcome of patients treated with IVrt-PA, and may be a better predictor of post-thrombolytic functional outcome in patients with previous antiplatelet therapy than in non-used patients.

## Introduction

Cerebral infarction is a cerebrovascular disease that threatens human health and life with high morbidity, high disability, and high mortality, and is the leading cause of death in Chinese population [1]. Atherosclerosis, especially the formation of carotid atherosclerotic plaque, is an important factor leading to the occurrence of ischemic stroke, and the roles of biomarkers that can predict the occurrence and outcome of ischemic stroke are crucial [2, 3]. The baseline level of CRP can predict the clinical outcome of cardiovascular diseases, such as angina pectoris, myocardial infarction, etc. [4, 5]. Data indicate that changes in the baseline level of inflammatory biomarkers may affect the acute phase of inflammation and clinical prognosis [6, 7]. However, there is very little study on the relationship between acute inflammation biomarkers and post-stroke functional outcomes. A potential prognostic biomarker of ischemic stroke is CRP, which is currently used to assess pathological inflammation and the progression of atherosclerosis [8, 9]. Since coronary atherosclerotic heart disease and atherosclerotic cerebral infarction share the similar vascular pathophysiology, CRP levels after stroke may also have prognostic value in clinic [10]. Patients of ischemic stroke with increased inflammatory factors in the circulatory system at admission have a higher mortality rate after admission [11]. Other studies have shown that a time-dependent inflammatory cytokines can predict stroke outcomes, and markers of adaptive immune function also affect functional outcome [12]. Meanwhile, the patient's baseline innate immune level may influence the adaptive immune mechanism during the neurological rehabilitation period [13]. Data have shown that the immune response after stroke is time-based as well, and the innate immune response occurs in the first 24 h after ischemic injury [14]. Therefore, CRP level measured within 24 h after stroke onset may be independently related to long-term prognosis of ischemic stroke [15]. In the present study, we used pre-thrombolytic CRP values as baseline levels to investigate the correlation between CRP and long-term functional prognosis in patients receiving thrombolytic therapy.

## Methods

### Study population and data collection

The present study performed a retrospective analysis of prospectively collected data from the Stroke Center of the First Hospital Affiliated of Soochow University between August 2016 and July 2018. All enrolled patients suffered from acute cerebral ischemic stroke within 4.5 h from symptom onset, treated with IVrt-PA, and were diagnosed of large-artery atherosclerosis (LAA) subtype at admission. LAA was defined as significant (≥ 50%) stenosis of a major artery relevant to the acute infarction, according to the Trial of ORG 10172 in Acute Stroke Treatment (TOAST) classification [16]. Patients who treated with endovascular mechanical thrombectomy, or had premorbid modified Rankin scale (mRS) score > 2 or had unavailable clinical and imaging information were excluded. All patients gave informed consent to join in and all data were analyzed anonymously. All patients received antiplatelet therapy daily for at least 3 months after onset of stroke. Ethical approval for this study was obtained from the ethics committees of the First Hospital Affiliated to Soochow University.

We collected socio-demographic characteristic, laboratory data and imaging information at admission and during hospitalization. Socio-demographic information on age, gender were collected. Lifestyle factors including smoking and alcohol consumption, past medical history, family history, disease history of hypertension, diabetes, stroke and coronary heart disease were obtained. Admission systolic blood pressure (SBP), admission diastolic blood pressure (DBP) were also systematically recorded. We also recorded laboratory data including total cholesterol (TC), low-density lipoprotein cholesterol (LDL-C), high-density lipoprotein cholesterol (HDL-C), triglyceride (TG), uric acid, creatinine, fibrinogen (FBG) and so on. All patients underwent routine EEG twice (on admission and before discharge) or Holter to exclude atrial fibrillation during the hospitalization. MIStar, the automatic software (Apollo Medical Imaging Technology, Melbourne, Australia) was used to calculate infarct core volume.

### Clinical assessment

All patients were evaluated separately by two neurologists on the basis of the National Institutes of Health Stroke Scale (NIHSS) score. NIHSS score was used to assess the severity of neurological deficit. Neurological functional outcomes at 3 months were determined based on the modified Rankin Scale (mRS). mRS > 2 represented poor clinical outcome. The follow-up was conducted by 2 trained neurological doctors who were blinded to the baseline information.

Alberta Stroke Program Early CT Score (ASPECTS) was used to evaluate early ischemia and collateral circulation [17]. The ASPECTS was determined from two standardized axial CT cuts, one adjacent to the most superior margin of the ganglionic structures and one at the level of the thalamus and basal ganglion. The total points were 10 points. When an area of early ischemic change appearance, a single point was subtracted. The early ischemic changes included focal swelling or parenchymal hypoattenuation.

The GWTG-Stroke sICH risk “GRASPS” score provides clinicians with a validated method to determine the risk of sICH in patients of ischemic stroke treated with rt-PA [18]. The total risk score was with a range of 45 to 101 points. The score consisted of 6 clinical predictor variables: increasing age (17 points), male sex (4 points), Asian race (9 points), high glucose at presentation (8 points), high systolic blood pressure at presentation (21 points) and NIHSS score (42 points).

### Statistical analysis

Continuous variables were expressed as mean ± standard deviation (SD) when data accorded with normal distribution. Otherwise, data were expressed in terms of quartile. Data were compared using an unpaired, 2-tailed t test or Mann–Whitney test. Categorical variables were compared using *χ*^2^ test or Fisher’s exact test. A segmented regression model was used to examine the threshold effect of hsCRP on mRS through spline smoothing. We also applied a likelihood model to compare the one-linear regression model with a two-piecewise linear model. The odds ratios (ORs) and 95% CIs of mRS in response to different exposure variables across therapy stratification were estimated. The final model retained selected factors to develop a ROC curve to discriminate patients with poor outcome (mRS > 2). The 95% CIs with concordance index (C index) were estimated using the bootstrapping method (500 iterations). All analyses were performed using the statistical package R version 3.6.3 (http://www.r-project.org/).

## Results

### Stratified analyses of studied population

**Fig. 1:**
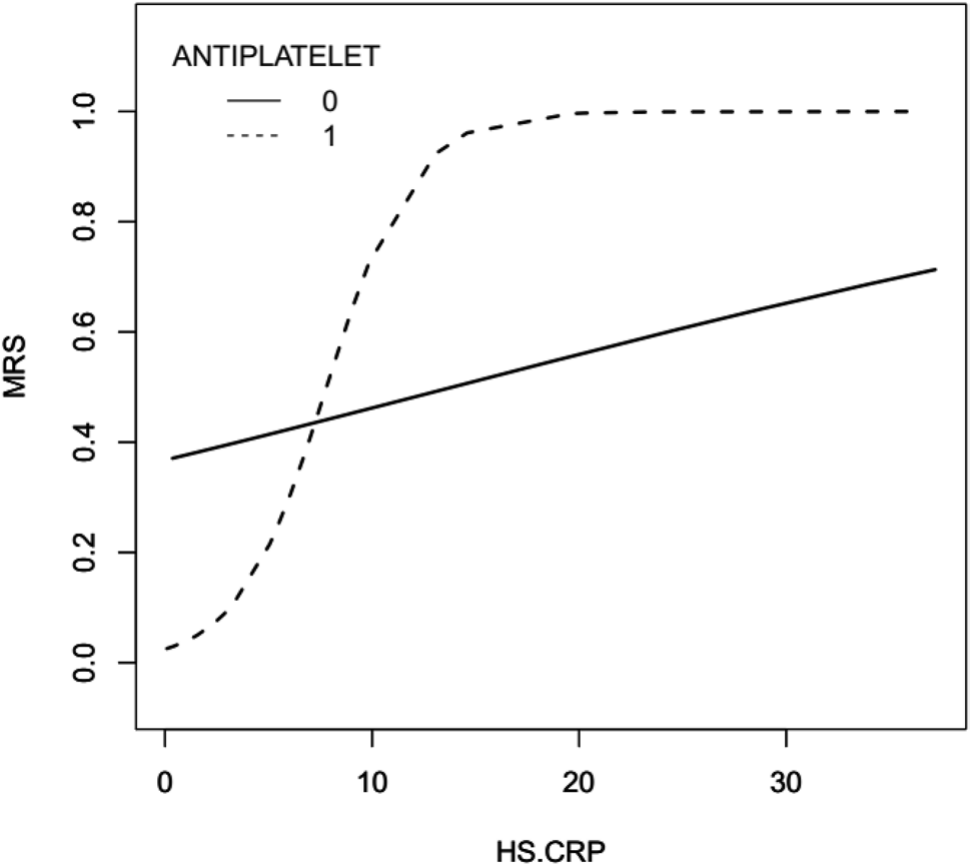
hsCRP was positively associated with long-term neurological functional outcome. Smoothing spline plots of mRS by hsCRP in therapy-stratified patients. Dashed line represents the patients with previous antiplatelet therapy, and solid line represents the patients without previous antiplatelet therapy

We recruited 194 eligible patients in this study. When analyzed using segmented regression model, the hsCRP was positively correlated with mRS in therapy-stratified analyses. There was a linear relationship between hsCRP and mRS in patients with non-antiplatelet history. By contrast, a non-linear relationship between hsCRP and mRS was found in patients with previous antiplatelet therapy. The threshold and saturation effect of hsCRP on mRS from piecewise linear regression is presented in Fig. 1. We further conducted dichotomous stratified analyses by therapy history. As shown in Table 1, in the patients without antiplatelet therapy, there were statistical differences of the cohort’s characteristics in age, NIHSS, GRASPS, infarct volume, DBP, previous hypertension, and ASPECTS. In the patients with previous antiplatelet therapy, the statistical differences were among age, hsCRP, creatinine, TG, infarct volume, GRASPS, FBG, and previous stroke (Table 2, *P* < 0.05).

**Table 1:** Comparison of characteristics in outcome-stratified patients without previous antiplatelet therapy

Characteristic	Mean (SD) or median (Q1–Q3)/*N* (%)		*P*-value
Non-antiplatelet history	mRS ≤ 2 (*n* = 93)	mRS > 2 (*n* = 70)	
Age	63.82 ± 12.11	68.71 ± 10.18	0.007
HS.CRP	3.40 (1.27–9.36)	4.29 (1.75–13.30)	0.063
Creatinine	71.83 ± 15.45	68.86 ± 19.45	0.279
Uric acid	306.28 ± 98.39	310.28 ± 91.72	0.792
Platelet count	199.08 ± 52.99	185.09 ± 50.81	0.091
NIHSS	4.00 (2.00–8.00)	10.00 (6.00–15.00)	< 0.001
TC	4.31 ± 0.91	4.65 ± 1.69	0.101
TG	1.22 (0.84–1.61)	1.30 (0.96–1.56)	0.213
HDLC	1.14 (1.01–1.26)	1.19 (1.07–1.41)	0.124
LDLC	2.53 ± 0.71	2.66 ± 0.70	0.236
VOLUME	1.98 (0.39–11.23)	6.17 (1.22–56.35)	< 0.001
GRASPS	70.84 ± 6.64	76.07 ± 8.18	< 0.001
SBP	150.46 ± 19.72	150.76 ± 23.74	0.931
DBP	85.39 ± 11.24	90.34 ± 20.15	0.048
FBG	5.61 (4.76–6.75)	6.01 (5.31–6.93)	0.054
Hypertension			0.006
No	42 (45.16)	17 (24.29)	
Yes	51 (54.84)	53 (75.71)	
Diabetes			0.488
No	73 (78.49)	58 (82.86)	
Yes	20 (21.51)	12 (17.14)	
Coronary heart disease			0.773
No	91 (97.85)	68 (97.14)	
Yes	2 (2.15)	2 (2.86)	
Stroke			0.161
No	87 (93.55)	61 (87.14)	
Yes	6 (6.45)	9 (12.86)	
Gender			0.183
No	23 (24.73)	24 (34.29)	
Yes	70 (75.27)	46 (65.71)	
Self-transferring			0.083
No	77 (82.80)	50 (71.43)	
Yes	16 (17.20)	20 (28.57)	
ASPECTS			< 0.001
7	0 (0.00)	5 (7.14)	
8	3 (3.23)	3 (4.29)	
9	14 (15.05)	26 (37.14)	
10	76 (81.72)	36 (51.43)	
HF			0.628
No	89 (95.70)	68 (97.14)	
Yes	4 (4.30)	2 (2.86)	
Smoking			
No	68 (73.12)	46 (65.71)	0.308
Yes	25 (26.88)	24 (34.29)	

Data are presented as mean ± standard deviation values or *n* (%)

*CRP* C-reactive protein, *NIHSS* National Institutes of Health Stroke Scale, *TC* total cholesterol, *TG* total glyceride, *HDLC* high-density lipoprotein cholesterol, *LDLC* low-density lipoprotein cholesterol, GRASPS, *SBP* systolic blood pressure, *DBP* diastolic blood pressure, *ASPECTS* Alberta Stroke Program Early CT Score, *HF* heart failure

**Table 2:** Comparison of characteristics in outcome-stratified patients with previous antiplatelet therapy

Characteristic	Mean (SD) or median (Q1–Q3)/*N* (%)		*P*-value
Prior antiplatelet	mRS ≤ 2 (*n* = 9)	mRS > 2 (*n* = 22)	
Age	65.33 ± 10.25	73.05 ± 8.20	0.035
HS.CRP	2.32 (1.16–5.38)	8.32 (6.80–18.35)	< 0.001
Creatinine	60.01 ± 14.59	77.98 ± 21.33	0.029
Uric acid	297.63 ± 75.04	288.79 ± 75.33	0.768
Platelet count	180.22 ± 55.86	160.50 ± 37.87	0.262
NIHSS	6.00 (3.00–6.00)	8.00 (5.25–14.50)	0.155
TC	4.21 ± 1.30	4.30 ± 0.78	0.808
TG	1.60 ± 0.39	1.20 ± 0.49	0.038
HDLC	1.11 ± 0.26	1.16 ± 0.32	0.688
LDLC	2.11 (1.77–2.58)	2.24 (2.02–3.14)	0.601
VOLUME	1.92 (0.43–12.05)	32.79 (8.82–79.54)	0.012
GRASPS	71.67 ± 6.61	78.27 ± 8.53	0.047
SBP	158.56 ± 11.04	160.00 ± 17.09	0.817
DBP	87.22 ± 11.03	90.64 ± 14.28	0.527
FBG	5.65 ± 0.82	7.35 ± 2.37	0.046
Hypertension			0.244
No	0 (0.00)	3 (13.64)	
Yes	9 (100.00)	19 (86.36)	
Diabetes			0.694
No	6 (66.67)	13 (59.09)	
Yes	3 (33.33)	9 (40.91)	
Coronary heart disease			0.054
No	9 (100.00)	15 (68.18)	
Yes	0 (0.00)	7 (31.82)	
Stroke			0.004
No	1 (11.11)	15 (68.18)	
Yes	8 (88.89)	7 (31.82)	
Gender			0.505
No	4 (44.44)	7 (31.82)	
Yes	5 (55.56)	15 (68.18)	
Self-transferring			0.062
No	7 (77.78)	9 (40.91)	
Yes	2 (22.22)	13 (59.09)	
*ASPECTS*			0.259
7	0 (0.00)	1 (4.55)	
8	0 (0.00)	3 (13.64)	
9	5 (55.56)	5 (22.73)	
10	4 (44.44)	13 (59.09)	
HF			0.849
No	8 (88.89)	19 (86.36)	
Yes	1 (11.11)	3 (13.64)	
Smoking			
No	6 (66.67)	9 (40.91)	0.193
Yes	3 (33.33)	13 (59.09)	

Data are presented as mean ± standard deviation values or *n* (%)

*CRP* C-reactive protein, *NIHSS* National Institutes of Health Stroke Scale, *TC* total cholesterol, *TG* total glyceride, *HDLC* high-density lipoprotein cholesterol, *LDLC* low-density lipoprotein cholesterol, GRASPS, *SBP* systolic blood pressure, *DBP* diastolic blood pressure, *ASPECTS* Alberta Stroke Program Early CT Score, *HF* heart failure

### Modification effects of antiplatelet therapy on the associations with neurological functional outcome

We further estimated the ORs (95% CI) for the strength of association of hsCRP/infarct volume and mRS in stratified analyses by antiplatelet therapy. Regression models were analyzed for crude, adjusted for all variables. The corresponding ORs and 95% CI are depicted in Table 3. Comparisons of interaction effects were presented between hsCRP and infarct volume. There was a statistical modification effect of antiplatelet therapy on the association of hsCRP and mRS (Table 3, *P* = 0.0021, and *P* = 0.0101, respectively). By contrast, there was no statistical modification effect of antiplatelet therapy for the association of infarct volume and mRS (Table 3, *P* = 0.1326, and *P* = 0.0682, respectively).

**Table 3:** Modification effects of therapy on the association between mRS and different exposure variables

	ORs (95% CI) *P* value	
	Crude	Adjusted
HS.CRP		
Without previous antiplatelet	1.04 (0.99, 1.09) 0.0957	0.96 (0.89, 1.02) 0.2112
With previous antiplatelet	1.60 (1.09, 2.36) 0.0164	1.34 (0.92, 1.95) 0.1277
*P* for interaction	0.0021	0.0101
Infarct volume		
Without previous antiplatelet	1.02 (1.01, 1.03) 0.0008	1.00 (0.99, 1.02) 0.5054
With previous antiplatelet	1.05 (1.00, 1.12) 0.0728	1.06 (0.99, 1.13) 0.1075
*P* for interaction	0.1326	0.0682

### ROC analyses of predicting neurological functional outcome

**Fig. 2:**
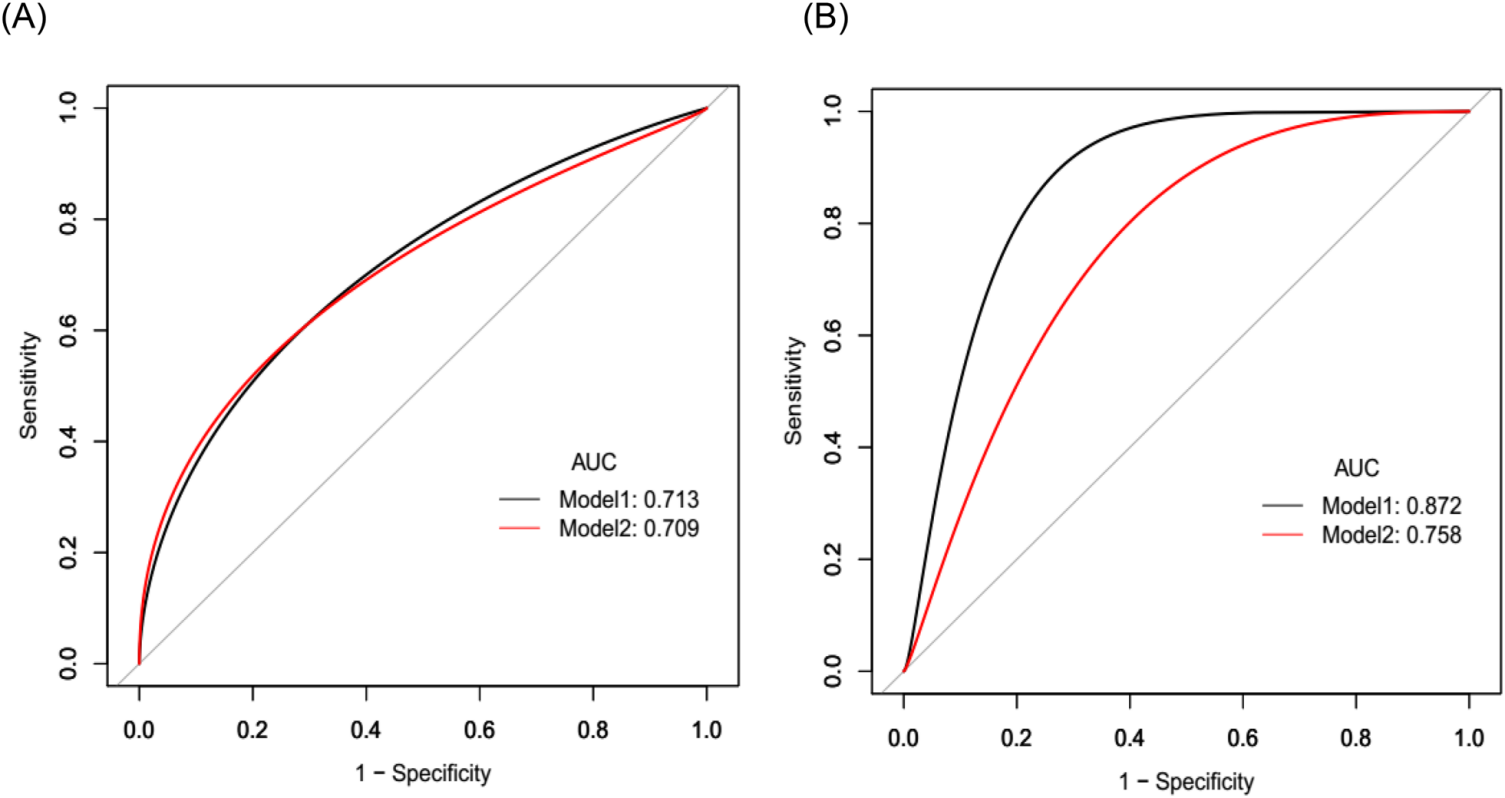
**A** Receiver operating characteristic (ROC) curve analyses of integrative variables (age, hsCRP, infarct volume, GRASPS, FBG, self-transferring) to discriminate patients (without previous antiplatelet therapy) with poor functional outcome. Area under the ROC curve (AUC) for model 1, hsCRP rule in was 0.7133 (95% CI 0.6326–0.7895), and for model 2, hsCRP rule out was 0.7087 (95% CI 0.6335–0.7810). **B** ROC analyses of integrative variables (age, hsCRP, infarct volume, GRASPS, FBG, self-transferring) to discriminate patients (with previous antiplatelet therapy) with poor functional outcome. AUC for model 1, hsCRP rule in was 0.8724 (95% CI 0.5556–0.9302), and for model 2, hsCRP rule out was 0.7582 (95% CI 0.4767–0.9133)

The variables with *P* value < 0.1 in both univariate analyses (age, hsCRP, infarct volume, GRASPS, FBG, self-transferring) were retained in the final analysis to develop an integrative predictive model to discriminate patients with poor outcome (mRS > 2). Two ROC models adjusted for the resulting factors (model 1, hsCRP rule in, and model 2, hsCRP rule out), with bootstrap validation were applied and compared (Fig. 2A, B). The predictive performance was improved as evidenced by a higher AUC in patients with previous antiplatelet therapy (from 0.758 to 0.872), whereas the weight of hsCRP in patients without antiplatelet therapy were lower (from 0.713 to 0.709).

## Discussion

CRP is a non-specific acute-phase protein mainly produced by liver smooth muscle cells and adipocytes under the stimulation of a variety of inflammatory factors. As a disease predictor, its serum level can rise rapidly with the stimulation of tissue injury, infection, inflammation and tumor proliferation [19]. At the same time, vascular smooth muscle cells and macrophages in plaque can also synthesize a small amount of CRP, which plays an important role in the chronic inflammatory process of atherosclerotic plaque formation [20]. The content of CRP in normal human serum is very low. In pathological conditions, however, e.g., cerebral infarction, CRP level has been found to increase during the first three hours in brain, reaching the peak at 48–72 h, and decreases after pathological process recovers [21, 22]. High-sensitivity C-reactive protein is a stable protein with long half-life and little diel fluctuation [23]. Its sensitivity and accuracy are higher than CRP, and is not affected by food and other external factors [24]. It is one of the most sensitive indicators to reflect the levels of inflammation, and has been widely used in clinic nowadays.

Consistent with the previous studies, our study indicated that the prognosis of patients with atherosclerotic cerebral infarction becomes worse as the basal hsCRP level increases. Previous studies have suggested that hsCRP may be an “incidental phenomenon” of inflammation in atherosclerosis, whereas not directly cause atherosclerosis. However, more and more evidence have suggested that hsCRP activates complement system and promotes neutrophil adhesion and aggregation, which may be an independent risk factor in atherosclerotic diseases [25, 26]. The expression of hsCRP is found in the atherosclerotic plaques, which activates macrophages to combine with oxidized low-density lipoprotein cholesterol, and then transforms into foam cells. Meanwhile, it stimulates the production of prethrombotic tissue factor and endothelial cells express adhesion molecules, resulting in abnormal endothelial function, and lead to the instability of atherosclerotic plaque [27]. Recently, it was found that the conformational reorganization of hsCRP may require its pro-inflammatory behavior, indicating a direct role of hsCRP to response in an inflammatory arteriosclerosis process with arterial walls [28]. A study involving 817 patients has showed that increased levels of hsCRP, IL-6 and fibrinogen were significantly associated with the increased incidence of vascular occlusive events, vascular mortality and non-vascular causes of death [29]. Three months after the onset of ischemic stroke, hsCRP and plasminogen activator inhibitor-1 can predict the progression of atherosclerosis in large intracranial arteries [30]. It has also been shown that hsCRP, IL-6 and protein-binding acrosomal proteins are risk factors for cervical arteriosclerosis [31]. Therefore, the measurement of hsCRP levels may be an effective predictor for patients with severe carotid artery stenosis [32].

Our primary finding is, to the best of our knowledge, the first to report that the predictive value of CRP for the clinical prognosis in patients who have previously received antiplatelet therapy is greatly improved. Platelets and inflammatory biomarkers have a synergistic effect in the process of atherosclerosis and thrombosis. Under pathological conditions, exposed collagen, invading pathogens, and inflammatory mediators can stimulate platelet activation [33]. Activating platelets produces two key effectors, namely, membrane surface receptor expression (e.g., Toll like receptor, TLR) and degranulation (e.g., IL-1β) [34]. The functional proteins act on effect cells in a paracrine manner, and on the other hand, the autocrine manner stimulates the continuous activation of platelets [35]. Therefore, it is speculated that the clinical benefits of antiplatelet therapy may be derived from the dual effects of antiplatelet and anti-inflammatory [36]. Antiplatelet drugs can be divided into cyclooxygenase inhibitors and P2Y12 receptor antagonists according to their mechanism. Among them, aspirin and clopidogrel are the most commonly used drugs. Aspirin inhibits the interaction of white blood cells and endothelial cells, and reduces the number of polymorphic neutrophils through the NO pathway, thereby inhibiting the inflammatory response. Unlike the controversial mechanism of aspirin, it is generally believed that P2Y12 receptor antagonists have anti-inflammatory effects [37]. It has been found in animal models that clopidogrel can also reduce the levels of pro-inflammatory factors such as TNF-α, IL-6, and chemokines in endotoxemia in brain [38, 39]. In a study of human systemic inflammation model, it is found that P2Y12 receptor antagonists can reduce platelet aggregation, reduce the peak of pro-inflammatory factors, and inhibit the production of d-dimer [40]. Compared with clopidogrel, ticagrelor can reduce the peak of IL-8 and the production of colony stimulating factor, and increase the level of anti-inflammatory factor IL-10 [40]. Thus, we speculate that patients whose baseline levels of hsCRP were still higher with previous antiplatelet therapy may have poorer pharmacogenetic sensitivity or adherence to the drugs when compared with patients at lower baseline levels. Therefore, patients whose baseline levels of hsCRP are still high with previous antiplatelet therapy require more precision secondary prevention.

Our study has several limitations. The data were validated with an observational study, and the threshold of hsCRP may differ when other confounders are controlled in randomized controlled trials. Therefore, inferences derived from our results may lack generalizability and maybe only applicable to specific populations similar to our study population. Thresholds require further studies with larger sample sizes using randomized methods. Other limitations of this study include missing data for some important variables, such as lack of other inflammation markers, lack of regular follow-up after discharge, lack of further evaluation of clinical outcome, and lack of record of the patient’s pharmacogenetic polymorphism or adherence. Finally, the single-center design may limit the generalizability of this conclusion.

In conclusion, hsCRP is highly correlated with the clinical prognosis of patients treated with IVrt-PA. hsCRP may be a better predictor of post-thrombolytic functional outcome in patients with previous antiplatelet therapy than in non-used patients.

## Data Availability

Data that support the findings of this study are available from the corresponding author upon reasonable request.
